# Exploring the effects of glucagon-like peptide-1 receptor agonists on ventricular arrhythmias: a propensity score-matched analysis

**DOI:** 10.1093/europace/euaf090

**Published:** 2025-05-02

**Authors:** Min Choon Tan, Aravinthan Vignarajah, Yong Hao Yeo, Justin Z Lee, Andrea M Russo, Luis R Scott, Dan Sorajja

**Affiliations:** Department of Cardiovascular Medicine, Mayo Clinic Arizona, 5777 East Mayo Boulevard, Phoenix, Arizona 85054, USA; Department of Medicine, Cleveland Clinic Fairview Hospital, Cleveland, OH, USA; Department of Internal Medicine/Pediatrics, Beaumont Health, Royal Oak, MI, USA; Department of Cardiovascular Medicine, Cleveland Clinic, Cleveland, OH, USA; Department of Cardiovascular Medicine, Cooper University Hospital, Camden, NJ, USA; Department of Cardiovascular Medicine, Mayo Clinic Arizona, 5777 East Mayo Boulevard, Phoenix, Arizona 85054, USA; Department of Cardiovascular Medicine, Mayo Clinic Arizona, 5777 East Mayo Boulevard, Phoenix, Arizona 85054, USA

**Keywords:** GLP-1 agonist, Ventricular arrhythmia, Outcomes

## Abstract

**Graphical Abstract:**
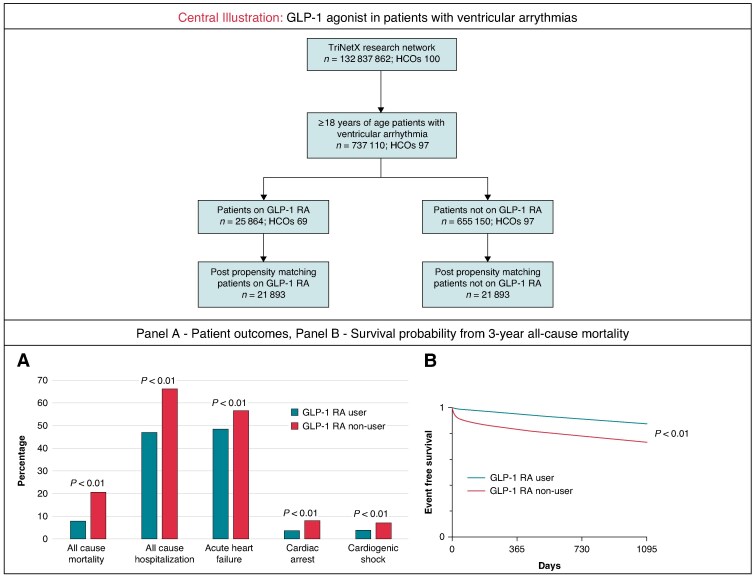

Despite advancements in the management of ventricular arrhythmias (VA), these cardiac conditions remain a leading cause of cardiovascular morbidity and sudden cardiac death, accounting for up to 300 000 deaths annually in the United States.^1^ Emerging clinical trial evidence has demonstrated a role for glucagon-like peptide-1 (GLP-1) receptor agonists in improving cardiovascular outcomes. However, their specific impact on patients with VA is not well established. Thus, we conduct this study to examine the possibility that GLP-1 agonists may have a role in managing VA by studying clinical outcomes of VA patients using a multi-national clinical database.

We performed a retrospective cohort study by analysing the TriNetX Analytics Research Network. TriNetX is a globally federated health research network using electronic health record data from >100 million patients. Patients aged ≥18 years with diagnosis of VAs (ventricular tachycardia, ventricular fibrillation) and diabetes mellitus/overweight/obesity were identified by *International Classification of Diseases, Tenth Revision* (*ICD-10*) from 1 January 2012 to 1 January 2021. We further categorized the patient population into 2 groups: those who had been on GLP-1 receptor agonists for at least 1 year and those who had not been on GLP-1 receptor agonists. Patients were 1:1 propensity score matched for patient demographics, BMI, cardiovascular medications, and 20 different cardiovascular comorbidities. Falsification endpoints such as clavicle fracture, acute conjunctivitis, and gastroenteritis were analysed to validate our findings. TriNetX uses a nearest-neighbor matching with a calliper of 0.1 pooled SDs. The study outcomes included 3-year rates of all-cause mortality, all-cause hospitalization, acute heart failure, cardiac arrest, and cardiogenic shock. Statistical analysis was performed using the TriNetX platform, with significance set at *P* < 0.05 (2-sided). TriNetX calculates odd ratios (ORs) and CIs using multivariate logistic regression. Survival analysis was conducted by plotting Kaplan–Meier curves and comparing the two cohorts with log-rank tests. This study does not require Institutional Review Board review nor informed consent because data are de-identified.

A total of 737 110 patients were identified, including 25 864 patients who were GLP-1 agonist users (3.5%). After propensity-score matching, 21 893 matched GLP-1 agonist users (62.5 ± 12.2 years of age, 41.7% female, 68.9% White, 20.6% African American, 6.9% Hispanic, LVEF 51.3 ± 15.3%) and non-users (63.2 ± 14.0 years of age, 41.6% female, 69.0% White, 20.7% African American, 6.9% Hispanic, LVEF 50.1 ± 16.2%) were analysed. Compared with non-users with VA, GLP-1 agonist users were associated with lower rates of 3-year all-cause mortality [odds ratio (OR): 0.331, 95% confidence interval (CI): 0.312–0.351] and all-cause hospitalization (OR: 0.456, 95% CI: 0.439–0.474). A lower risk of cardiac arrest (OR: 0.415, 95% CI: 0.381–0.451), acute HF (OR: 0.723, 95% CI: 0.696–0.751), and cardiogenic shock (OR: 0.528, 95% CI: 0.485–0.575) were also observed in GLP-1 agonist user cohort during the 3-year follow-up duration. Additional analysis among patients with VT and those with VF also showed similarly reduced odds of cardiac adverse events. There was no significant difference in falsification endpoints (acute clavicle fracture: OR: 0.821, 95% CI: 0.583–1.156; acute conjunctivitis: OR: 1.468, 95% CI: 0.922–2.335; acute gastroenteritis: OR: 0.902, 95% CI: 0.727–1.120) between the two cohorts.

This study provides crucial insight into the 3-year outcomes observed among patients with VA who were on GLP-1 agonist therapy. Our study found that the use of GLP-1 agonist therapy is associated with a lower risk of all-cause mortality, hospitalization, cardiac arrest, acute HF, and cardiogenic shock in this high-risk population. The observed benefits could be attributed to the unique action of GLP-1 agonist, including the ability to stabilize cardiac electrical conduction, and the anti-inflammatory and anti-fibrotic effects.^2^ However, it is important to highlight that a causal relationship cannot be established due to the nature of our study design and the lack of granular data, such as baseline patient profiles between cohorts within our database. With all the limitations of an observational design, these findings suggest that the incorporation of GLP-1 agonists into the management of VA, may be useful to optimize cardiovascular outcomes in this vulnerable patient group, even if further confirmation will be needed. In addition, it is important to emphasize that disease-specific management and established therapeutic strategies, including anti-arrhythmic drugs, implantable cardioverter-defibrillators, and catheter ablation remain the cornerstone of VA and sudden cardiac death management.^3-6^

There are several limitations in our study. First, as with most of the large administrative database studies, the main limitation includes miscoding in primary diagnoses and under-reporting of secondary diagnoses. Additionally, miscoding of the assessed outcomes may lead to inaccurate result. Second, the database has no granular procedural data, such as the duration and adherence to GLP-1 agonist therapy, the extent of weight loss, the length of VA events, and diabetes control status, which restricts our ability to fully understand the mechanisms behind the observed outcomes. Third, data on the exact cause of mortality are not available in the database, limiting our understanding of the observed mortality benefit.

In conclusion, our study found a possible association between GLP-1 agonist use and more favourable outcomes in patients with VA, suggesting a potential therapeutic role for this medication in these high-risk patients. However, further prospective studies are needed to explore and confirm these findings.

## Data Availability

The data sets generated and analysed in this study are available from the corresponding author on reasonable request.
